# Recent progress in the conversion of biomass wastes into functional materials for value-added applications

**DOI:** 10.1080/14686996.2020.1848213

**Published:** 2020-12-14

**Authors:** Chufan Zhou, Yixiang Wang

**Affiliations:** Department of Food Science and Agricultural Chemistry, McGill University, Quebec, Quebec, Quebec, Canada

**Keywords:** Biomass wastes, recycling, functional materials, value-added applications, 104 Carbon and related materials, 100 Materials

## Abstract

The amount of biomass wastes is rapidly increasing, which leads to numerous disposal problems and governance issues. Thus, the recycling and reuse of biomass wastes into value-added applications have attracted more and more attention. This paper reviews the research on biomass waste utilization and biomass wastes derived functional materials in last five years. The recent research interests mainly focus on the following three aspects: (1) extraction of natural polymers from biomass wastes, (2) reuse of biomass wastes, and (3) preparation of carbon-based materials as novel adsorbents, catalyst carriers, electrode materials, and functional composites. Various biomass wastes have been collected from agricultural and forestry wastes, animal wastes, industrial wastes and municipal solid wastes as raw materials with low cost; however, future studies are required to evaluate the quality and safety of biomass wastes derived products and develop highly feasible and cost-effective methods for the conversion of biomass wastes to enable the industrial scale production.

## GRAPHICAL ABSTRACT

**Figure:**
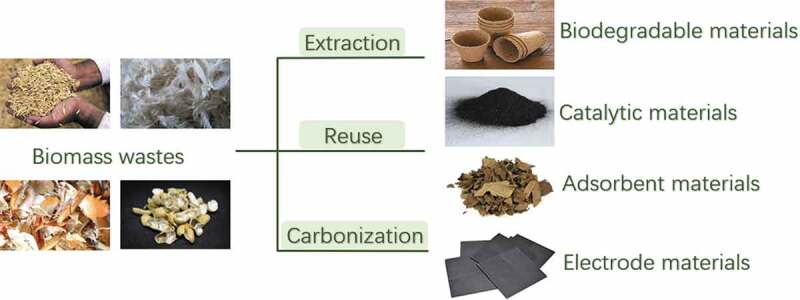

## 1. Introduction

The disposal, utilization and management issues of biomass wastes are a burgeoning challenge to cities especially in developing countries due to the increasing generation of biomass wastes and the lack of feasible and efficient approaches to recycling these biomass waste resources. Therefore, the rational reuse of them has attracted much attention around the world [1–3]. Biomass is a stored source of solar energy initially collected by plants via the process of photosynthesis where CO_2_ is captured and transferred to plant materials. It covers ranges of organic materials derived from plants and animals fed on the plant sand [4], and it can be converted into a variety of useful sources of bioenergy [5]. Nowadays, biomass energy has become the world’s fourth largest energy source, following coal, oil and natural gas [1].

Biomass wastes commonly consist of forestry residues, agricultural wastes, animal wastes, industrial wastes, municipal solid wastes (MSW), food processing wastes and so on [3,6]. Among various kinds of biomass wastes, biomass feedstock from agricultural and forestry wastes, animal wastes, industrial wastes, and MSW have been widely investigated in recent five years to extract natural polymers or to be converted into functional materials for value-added applications. Majority of biomass wastes are left in the field to naturally decompose, or discarded in landfill, or incinerated in the open for cooking, drying, and charcoal production, which not only have low efficiency, but also lead to severe environmental pollution like greenhouse gas emissions and air quality deterioration. Thus, converting biomass wastes into value-added products for various applications such as medicine, materials, and food packaging has been drawn attention recently.

Although several literature reviews have discussed the importance of recycling biomass wastes, most of them merely focus on a certain kind of biomass wastes, for example wood biomass [7], agricultural waste peels [8], biochar [9,10], activated carbon for adsorption of dye [11] and biomass waste cellulosic materials for nanocrystalline cellulose extraction [12]. Thus, this review systematically summarizes biomass wastes that have attracted most interest during the last five years. Biomass wastes are considered as biomass feedstock from the waste stream and are mainly derived from agricultural and forestry resources, animal resources, industrial resources and MSW. In one aspect, the recent research focuses on the recycling of natural polymers, such as cellulose, lignin, collagen, gelatin, keratin, and chitin/chitosan. They are extracted from various biomass wastes, including rice husk, peanut shell, corncob, head, skin, bones of fish, shrimp, crabs and wastepaper, and then used to fabricate functional materials with potential applications in biomaterials [13], coating for packaging [14], films for bioactive molecule delivery [15], water treatment [8], supercapacitor [16] and construction [10]. In another, biomass wastes could be directly applied, or converted into carbon-based materials as novel adsorbents, catalysts carriers, electrode materials, and functional composites.

## 2. Biomass wastes for functional material synthesis

In last five years, agricultural and forestry residues, animal wastes, industrial wastes, and MSW have been frequently studied. Most of them are under-utilized for energy and material production, which means only a small amount of biomass wastes can be used as a feedstock for real industrial applications [6].

### 2.1. Agricultural and forestry wastes

It is estimated that the generation of rice straw, wheat straw, corn straw, sugarcane bagasse, and rice husk, the most abundant biomass wastes from agricultural wastes, is 731, 354, 204, 181 and 110 million tons (Mt) per year, respectively [17]. In addition, the global production of wood biomass wastes is 4.6 Gt every year, with 20% being production loss [6]. Wastes from the olive oil industry are up to 30 Mt every year, and coffee industry produces 7.4 Mt of spent coffee grounds, coffee pulp, cherry hush [17], causing severe disposal problems and low efficiency of utilization.

#### 2.1.1. Crop straw

Crop straw is the dry stalk or stem produced in the field after removing the grain and chaff. The amount of crop straw is extremely huge all over the world. For example, the generation of crop straw in China has already reached about 600–800 Mt annually [18]. Burning is the commonest and cheapest method to eliminate crop straw for preparing land after harvesting corps, leading to the deteriorated air quality and other environmental problems [19]. Cellulose, hemicellulose, and lignin are the main constituents of crop straw. Crop straw is promising lignocellulosic raw materials to produce biofuel and some chemicals, but physico-chemical, bio and enzymatic pretreatments are needed due to its chemical constituents and the structure of lignocellulose. For example, biodiesel can be obtained by fast pyrolysis and catalytic liquefaction followed by hydrolysis, fermentation, and alkali catalysis processes [20]. The technology of producing fuel ethanol from straw mainly includes three steps: pretreatment, hydrolysis, and fermentation, among which are to degrade the carbohydrates in the plant cell wall and convert them into monosaccharides. On top of that, crop straw can be converted into biochar to immobilize metals [21].

#### 2.1.2. Rice husk

Rice husk is one of the most abundant agricultural waste materials that are ecofriendly and biodegradable. It can be separated from rice grain during the rice milling. Rice husk consists of cellulose (25–35%), hemicellulose (18–21%), lignin (26–31%) and silica (15–17%). Apart from these structured components, some other nonstructural components such as pectin, waxes, and inorganic salts are also included [22]. The global rice production was estimated at 769.7 Mt in 2017 [23]. As a result of such huge production of rice, millions of tons of rice husk are formed annually, leading to disposal problems. Many attempts have been made to recycle rice husk, such as used as fodder for animals, energy generation for heat, and electricity and biogas by combustion and gasification [24]. In recent five years, it is found that burning rice husk can produce different by-products such as rice husk ash and carbonized rice husk as sustainable additives to produce cement-based construction materials [23,25]. In addition, rice husk can be added into films as the filler, which can influence the properties of the filler-reinforced composites [26–28].

#### 2.1.3. Peanut shells

The peanut industry is the main source for the generation of peanut shells. According to the Food and Agriculture Organization of the United Nations report [29], peanut shells account for 20% of the whole peanut and the peanut production is up to approximately 46 Mt every year. Majority of peanut shells are discarded or burnt as wastes during the process or used as fodder for animals. When they are added into fodder, they cannot be used directly unless they are crushed and processed by proper chemical treatments because they contain some harmful chemicals and the lignin in peanut shells is difficult to be digested [30]. In one aspect, the disposal problem of peanut shells is getting worse; in the other, the high-energy content of peanut shells is worth exploring. Many works have focused on recycling and taking advantage of peanut shells. It has been reported that peanut shells contained a large amount of total polyphenol, flavonoid, and amino acid substances, which endowed them with superior antioxidant capacity and functional properties [31]. Moreover, the porous structure of peanut shells makes it potential to form gas-permeable packaging films for extending shelf life of food products [32]. It can also be utilized as carbon source for plant biomass-degrading enzymes, precursor for adsorbent to remove heavy metals [33], H_2_ production, bio-carbon production, construction materials, and so on [34,35].

#### 2.1.4. Corncob

Corncob is the central core of corns. It contains 39–45% of glucan, 25–35% of xylan, 17–21% of lignin, and low amounts of extractives [36]. Like the other agricultural wastes, corncob is usually incinerated for cooking and heating, causing serious environmental pollution. Recently, the researchers are considering new applications to explore corncob’s high-energy content. However, corncob needs to be pretreated or hydrolyzed to break the links among cellulose, hemicellulose, and lignin for further applications due to its complex composition and structure. For example, due to the high lignocellulosic constituents of corncob, chemicals such as furfural, xylooligosaccharides and glucose can be produced [37]. In addition, biochar can be obtained by the pyrolysis of corncob for energy applications [38].

#### 2.1.5. Others

In addition to the agricultural and forest wastes mentioned above, there are large numbers of other wastes in nature such as rattan, bagasse, and nutshells that have tremendous prospects for development. For example, shells from pecans, almonds, acorns, and bamboo have high porosity and are useful in the production of activated carbon by pyrolysis [39]. They can be applied as bio-fillers to reinforce thermoplastics via extrusion and injection molding processes as well [3]. In addition, the production of hydrogen from sunflower husk and bagasse and the conversion of levulinic acid to γ-valerolactone have been studied using Ni/NiO catalyst [40,41].

### 2.2. Animal wastes

Most animal wastes are generated from fishery, meat, leather and poultry industries such as fish/shrimp/crab wastes, animal and livestock manure, tannery wastes, feathers of chicken or other poultries, and so on. Among them, the seafood wastes, including shells, heads, skins, tails, fins, and bones, are the most abundant resources of biomaterials. During 2016–2017, the production of fish in India was up to 11.41 Mt, and 70% marine fish were processed before final sale, which left 20–80% fishing wastes [42]. In 2006, the total worldwide generation of shrimp reached 6 Mt, but only 60% were used as food, creating about 2.3 Mt of inedible wastes [43]. They were discarded nearby harbor or at the sea and were broken down by the aerobic bacteria living in the sea, resulting in the relative reduction of oxygen level under the sea [42]. In addition, environmental problems such as higher mortality of marine organisms due to the production of toxic H_2_S, spreading pathogens that may make an adverse effect on human health, and emitting bad fishy smell that contaminates fresh air are increasing. Currently, the seafood, skin, bones, exoskeletons, and viscera are the most explored [44], which are considered as the great potential sources to recover natural substances (e.g. collagen, gelatin, chitin, and chitosan).

On the other hand, poultry and meat industries produce large amounts of inedible portions such as bones, feathers, tendons, and skins with the fast-growing rate due to the higher demand of consumers. It was reported that the annual generation of feather and offal was already up to 8 × 10^5^ tonnes over the world [45]. Pathogens exist in these wastes, so it is essential to treat them as soon as possible. The common treatments are incineration or composting them with manure, but several problems occur such as high-energy consumption, emission of carbon dioxide, odorous smell of H_2_S from composting, and so on.

Due to the above-mentioned problems, the recent studies focused on developing sustainable high-value applications of animal waste, including extraction of biopolymers (e.g. collagen, keratin, and gelatin) [46], production of biogas [47], commercial crop (e.g. tomato) [48], and biodiesel [49].

### 2.3. Industrial wastes

Black liquor and industrial sludge sewage from manufacturing are main constituents of industrial biomass wastes. Black liquor is a major pollution source in pulp and paper mills. It consists of 65–85% solid content, containing inorganic matters from cooking chemicals (e.g. sodium hydroxide, sodium sulfide) in the digester of processing and organic materials (e.g. lignin) from lignocellulosic biomass [50]. It is estimated that each ton of pulp generated by Kraft pulping process can produce approximately 10 tons of black liquor [50], which accounts for 90% pollution of the pulp and paper industry [51]. Black liquor is traditionally treated to extract lignin by super-filtration, agglomeration and acid separation and recover alkali by combustion. However, these conventional methods have high operation cost and need huge pre-equipment investment [51]. Till now, many researchers have investigated the applications of black liquor derived products in functional fertilizer synergist [52], bio-fuel [53,54], and bio-plastic [50].

Industrial sewage sludge with high moisture content is generally produced from the industrial wastewater treatment facility. Because sewage sludge contains various hazardous matters, pathogens, heavy metals, and microplastics, it is difficult to properly treat it [55]. Excect from being recycled as fertilizer, new approaches have been proposed to generate valuable resources and materials from industrial sewage sludge over past five years, including the production of sludge pyrochar as adsorbent and soil conditioner [55], and sewage sludge ash for applications in heavy metal removal and construction of cement-based materials and cementitious binder [[56–58]].

### 2.4. Municipal solid wastes

Textile and wastepaper are major contributors to MSW with the generation of 16 Mt just in United States in 2014 for textile and 400 Mt globally for wastepaper [59]. However, the recycling of these wastes has not reached to a high rate. For example, textile accounts for about 5% of the landfill and global textile recycling is only about 13%, while recycling of wastepaper and cardboard stands for 58% [60]. The majority of wastes are discarded into landfill or incinerated, leading to negative impacts on the environment by contaminating ground water and generating greenhouse gases during decomposition [61]. Several attempts have been made to re-utilize wastepaper and cardboard, such as recycling of cellulose into antimicrobial packaging [59], producing biochar and bio-oil through pyrolysis [62] and obtaining cellulose nanocrystals [63]. In terms of textile made from a variety of materials, novel recycling approaches have also gained attention, including recovering polyester fibers and glucose syrup from waste polyester cotton blends [64], recycling cellulosic fibers from cotton wastes [65], and preparing insulation materials [61].

## 3. Extraction of natural polymers

**Figure 1.**
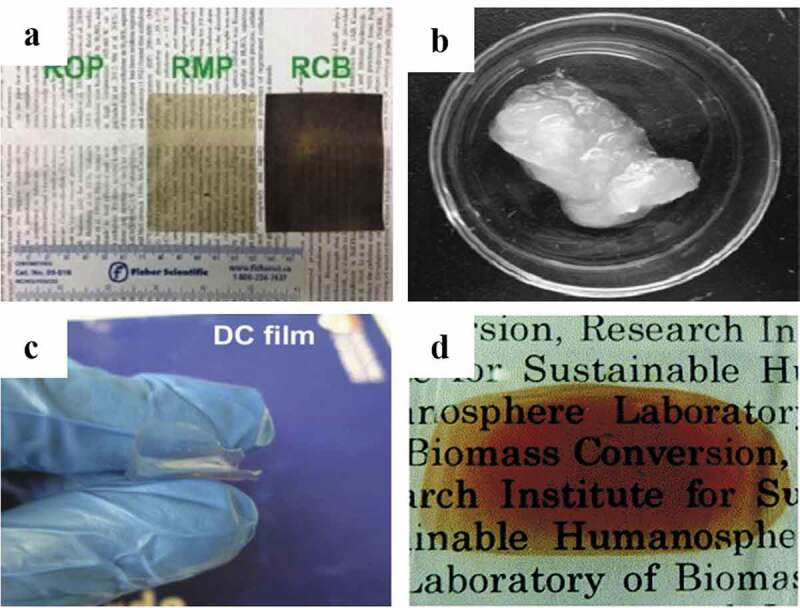
Films and hydrogels derived from different biomass wastes: (a) films from wastepaper; (b) cellulose nanofibril (CNF) hydrogel from waste sackcloth; (c) film from durian rind; and (d) film from *Eucalyptus globulus* wood chips [59, 79–81] (Reproduced by permissions from [80], copyright [2014, The Royal Society of Chemistry], from [59], copyright [2020, Elsevier], from [79], copyright [2015, Elsevier] and from [81], copyright [2019, Elsevier])

Most synthetic polymers are derived from fossil resources. The environmental and socioeconomic consequences have aroused much concern because many of synthetic polymers (e.g. polyethylene, polypropylene, and polystyrene) are non-renewable and non-biodegradable. Much attention has been paid to natural polymer-based materials, which are usually non-toxic, biodegradable, and biocompatible. Recently, a new trend has been found to recycle natural polymers from biomass wastes. The extraction of cellulose, lignin, collagen, gelatin, keratin, and chitin/chitosan from biomass wastes is summarized in Table 1. These natural polymers can be then used to prepare various films and hydrogels (Figure 1) for food packaging and biomedical applications.

**Table 1.** Extraction of natural polymers from biomass wastes

Polymer	Raw material	Extraction method	Extraction yield (%)	Purity
Cellulose	Coconut shell [13]	Organosolv extraction: reaction with ethanol/HNO_3_ followed by NaOH	70–95*	Free of lignin and hemicellulose
	Oil palm frond (OPF) [66]	Different concentrations of NaOH solution under pressure and non-pressure	n.d.	91.33%
	Areca nut husk [14]	Chemo-mechanical method: milling, homogenization, alkali treatment, acid hydrolysis, and bleaching	22–26**	85.47%
Lignin	Rice straw [67]	Alkaline (sodium hydroxide) and acidic (formic acid/acetic acid) treatment	n.d.	n.d.
	Corn stover [68]	Organic amine and organosolv synergetic pretreatment	81.7*	n.d.
	Sugarcane bagasse [69]	Ionic liquid	90.1*	Almost in pure form
Collagen	Skin/hide trimming wastes [70]	Propionic acid and acetic acid solubilization	93*	n.d.
	Bovine hide [71]	Acid-solubilization and acid-enzyme solubilization (AES)	n.d.	75.13%
	Chicken sternal cartilage [72]	Pepsin and ultra-sonication treatment	~80*	High purity
Gelatin	Atlantic mackerel skins [73]	Organic acids: acetic, citric, lactic, tartaric, or malic acid	29.6–31.8*	n.d.
	Camel skins [74]	Calcium hydroxide and ammonium sulfate	36.8–42.2*	88.21–90.42%
	Golden carp [75]	Prior-ultrasonication-acid treatment	62.12*	n.d.
Keratin	Chicken feathers [45]	Thermo-chemical treatments with different reducing agents	82–94*	n.d.
	Red sheep’s hair [76]	Sodium metabisulfite, urea with sodium dodecyl sulfate (SMBS)	96*	Higher than 90%
	Hog hair [77]	A two-step thermal hydrolysis process	68*	89.2%
Chitin Chitosan	Shells of crab, crayfish and shrimp [78]	Twice NaClO treatments before demineralization and deproteinization	n.d.	n.d.
	Shrimp shells [43]	Concentrated and diluted chloric acids, nitric acids, and sulfuric acids	n.d.	n.d.

*Calculated by the weight of extracted polymer/the weight of polymer in raw material **Calculated by dry weight of extracted polymer/dry weight of raw material n.d. Not determined

### 3.1. Cellulose

Cellulose is the most abundant polymer, representing approximately 40–50% of plant and woody biomass by weight [82]. Cellulose exhibits a high strength and is renewable and biodegradable, so it is widely used for fabricating optical films, coatings, and controlled released systems, as well as in textile and paper industries [83]. Mechanical, chemical, biological, enzymatic, and their combination treatments are common methods to extract cellulose from biomass wastes [84]. Substances like hemicellulose and lignin are removed by multistep treatments and cellulose is then obtained with high purity. Compared to single treatment, combination of mechanical, chemical, and biological treatments can increase the quality of cellulose; however, it increases the cost and causes high-energy consumption. For example, Kumneadklang et al. [66] treated OPF with NaOH solution with various concentrations under pressure of 7 bar and non-pressure (1.013 bar). It was found that α-cellulose fibers with highest content (91.33%) and crystallinity (77.78%) were obtained by using 15 wt.% NaOH at 150°C and 7 bar. Except from combination of treatments, it has been reported that organosolv process is a promising approach for the pretreatment of biomass wastes, because the lignocellulosic structure can be broken down and fragmented, and the constituents (e.g. cellulose, lignin, and hemicellulose) are subsequently isolated with high purity. For example, Amaral et al. [13] isolated cellulose from babassu coconut shells through organosolv with an acid catalyst, and the yield was about 70–95%. Comparison with conventional pulping processes (e.g. Kraft process and sulfite process) with yields of 50–60% [85], these new isolation processes are faster, more effective, and less harmful to the environment.

Apart from extracting cellulose by various methods, novel solvent systems have also been investigated recently to dissolve cellulose directly from biomass wastes, such as N-methyl morpholine-N-oxide (NMMO) [86], LiCl/N,N-dimethyl-acetamide (DMAc) [87], ionic liquids [88], alkali/urea [89] and sulfuric acid solvents [90]. Compared to traditional cellulose dissolution namely viscose process, these novel systems are eco-friendly, and effective. For example, Oliva et al. [59] dissolved wastepaper within only 210 seconds through pre-cooled H_2_SO_4_ aqueous solution and converted it into regenerated cellulose films with antimicrobial activity.

Additionally, celluloses with special structures such as CNF [79,91], cellulose nanocrystals (CNC) [92,93], and bamboo fibers [94] were also separated from textile waste, wastepaper, rattan, and so on. These nanocelluloses are well known as the reinforcing agents in building composite materials, textile engineering, musical instruments, food packaging, quality sensors, and drug delivery systems [3,92]. Cao et al. [79] developed H_2_O_2_/HNO_3_ solution as bleaching agent and hydrolysis medium to extract CNF from waste sackcloth. Non-cellulosic components were removed, and the obtained CNF possessed good dispersibility in different solvents. A chemo-mechanical method was developed to isolation of CNF from areca nut husk with the yield of 85.47% and the crystallinity of 73%. The chemical processes included alkali treatment, acid hydrolysis, and bleaching, while the mechanical treatment was involved in grinding and homogenization [14]. Ahuja et al. [91] extracted both highly purified lignin and crystalline CNF simultaneously from jute bag waste. Soda cooking pretreatment was applied to reduce recalcitrance of lignocellulose, and then hemicellulose was hydrolyzed into sugar, leaving cellulose and lignin residues. Huang et al. [92] reviewed the extraction and modification of CNCs from biomass wastes in the past five years and highlighted the potential applications of CNCs in food-related areas. It was found that although novel extraction methods have been developed, sulfuric acid hydrolysis was still applied widely.

### 3.2. Lignin

Lignin is the key structural material in the formation of plant cell walls, especially in wood and bark due to its aromatic structure that enhances the strength and rigidity [3]. The content of lignin in different plants varies. For example, in hardwood, grass and softwood, the lignin content is 20–25%, 10–15%, and 25–35%, respectively [3]. Generally, lignin is obtained as a by-product through paper pulping process, and the production was about several million tons over the past few years. However, it has low-value and is usually burnt for heat and power [95]. Therefore, significant attention has been paid to extract lignin from plant-based biomass wastes and used as antioxidant, absorbent, binder, dispersant, and so on, which mostly rely on its structure with hydrophobic constituents [96,97].

The complex structure of lignin makes it difficult to be isolated and increases the industry cost during the process. Lignin could present different physicochemical properties, which depend on the type of biomass and method of extraction [17]. Numerous methods have been applied for extracting lignin from biomass such as milled wood lignin (MWL), kraft pulping, sulfite pulping, soda process [17], organosolv process [68] and so on. The common way to derive lignin from wood is MWL, where plant materials are milled, and then extracted by dioxane and water. The disadvantage of this method is the low yield (10–60%) and structure change during the extraction process [98]. Kraft pulping and sulfite pulping are the traditional papermaking processes, while the soda process is suitable for the extraction of lignin with low molecular weight and high purity from non-woody biomass [17]. Except from frequently used pulping process, the organosolv process can isolate lignin with high purity under mild conditions in a more environmentally friendly way. This method solubilizes lignin and hemicellulose from lignocellulosic biomass into water or an organic solvent (e.g. ethanol, methanol, acetone, acetic acid, ethylene glycol, etc.), leaving an insoluble cellulose pulp for enzymatic hydrolysis [99]. According to selected organic solvents, catalysts may be used in the processes as well, including acids, bases, Lewis acids, and ionic liquids [99,100]. For example, an organic amine catalytic organosolv treatment was performed to obtain high-quality lignin from corn stover with the yield of 81.7% [68]. Ionic liquid 1-ethyl-3-methylimidazolium acetate ([EMIM]OAc) was also applied for the extraction of lignin from sugarcane bagasse with 90.1% yield [69]. Compare to traditional inorganic or solid base treatments, the solvent with catalyst in this system could be easily recycled and reused. However, these mono-phase solvents still require another processing step to separate lignin and hemicellulose [99]. Therefore, a two-phase system (water/1-butanol solvent) was introduced to extract lignin with a high quality [99,101]. Although these novel processes resulted in the high yield and purity, the relatively high cost and complicated operations limit their industrial-scale application.

### 3.3. Collagen

Collagen is the major constituent of collagenous solid wastes (e.g. skin, muscle, and tendons) generated from the fishing processing [42], leather trimming [70], poultry [46], and other industries. It is a fibrous, biocompatible and biodegradable protein, which is rich in animals and, accounts for about 30% of the body’s total protein [102]. There are several common methods to isolate collagen, including acid solution, acid solution with enzymes and neutral saline solutions [103]. For acid extraction, propionic acid solubilization approach could obtain collagen with higher yield (93%) from raw skin or hide trimming wastes [70], compared to acetic acid treatment with a yield of 85%. Another modified acid-enzyme solubilization method could extract collagen from cow hides with the highest yield of 75.13% [71]. Recently, several advanced technologies that are economical, effective and time saving have been springing up, including high pressure, electrical pulse, and ultra-sonication [72]. For example, Akram and Zhang [72] extracted collagen from chicken sternal cartilage through the ‘green’ method using pepsin and ultra-sonication treatments. The highest collagen-Ⅱ content was approximately 80%. In addition, this method was proved to improve the physicochemical and functional properties such as thermal stability of collagen-Ⅱ. To date, collagen derived from animal wastes has been applied in terms of tissue engineering, biomaterials, pharmaceutical and cosmetics industry [70]. Salim et al. [46] obtained collagen from chicken feet via a facile method of pepsin digestion and used it as a precursor to form low-cost porous carbon fibers in large quantities by wet spinning. Govindharaj et al. [42] treated waste eel skin with acetic acid, common salt and pepsin to obtain the collagen, which was then incorporated into alginate hydrogel to produce 3D scaffolds for tissue engineering application.

### 3.4. Gelatin

Gelatin, a kind of hydrocolloid and high molecular weight peptide that contains most amino acids, is usually derived from collagen. Factors like different extraction methods, extraction conditions (e.g. specifically the temperature, pH, pretreatments, and time), types of raw materials, and hydrolysis of collagen could relatively affect the yield, quality and functionalities of gelatin [74]. It has various applications in food industry (e.g. thickening agent, stabilizer, texturizer, and gelling agent), pharmaceutical industry and membrane technology because of its unique gel strength, rheological properties, and excellent film-forming ability [44]. Boughriba et al. [44] extracted gelatin from fresh blackchin guitarfish through NaOH treatments, while Khiari et al. [73] recovered gelatin from Atlantic mackerel skins by various organic acids. No significant difference was observed among these organic acids, and the highest yield was 31.8%. Al-Hassan [74] used calcium hydroxide and ammonium sulfate to isolate gelatin from the skins of camels, and the yield of gelatin was up to 36.8–42.2%. However, these conventional methods generally obtained a low yield because the linkages among collagen molecules are quite stable. Therefore, ultrasound treatment has attracted much attention, which could improve the extraction efficiency and the functional properties of gelatin. For instance, Ali et al. [75] pretreated golden carp skin with different acids, with or without prior-ultrasonication, to extract gelatin. It was found that the method with sulfuric acid/acetic acid along with prior-ultrasonication improved the efficacy of extraction (highest yield of 62.2%) and gelling strength of the obtained gelatin.

### 3.5. Keratin

Keratin is a significant component in hair, chicken or bird’s feathers, bristles, horns, hooves, and nails. Millions tonnes of keratin containing wastes are produced every year over the world, typically in poultry slaughterhouses and fabric textile industry [45]. The content of keratin in animal hair (including human hair) and poultry feathers is over 90% [104]. It is present as α-keratin in hair, horns and hoofs and β-keratin in bird feathers, respectively. Keratin plays an important role in providing a tough matrix for substances due to its mechanically durable property. The extraction, purification and characterization of keratin from raw materials can facilitate the development of keratin-based materials for tissue engineering [76], cosmetics, agriculture, and biodegradable packaging [45].

Soluble keratin can be obtained by hydrolysis (e.g. alkaline, acid, or enzyme), oxidation or reduction of disulfide bonds, thermal treatment, and steam flash explosion [45]. The yield of keratin through hydrolysis depends on temperature, pH, time, and type and concentration of alkaline, acid, and enzyme. Sinkiewicz et al. [45] used 2.5% NaOH pretreatment and thermo-chemical treatment with different reducing agents (e.g. 2-mercaptoethanol, dithiothreitol, sodium metabisulfite, and sodium hydroxide) to prepare keratin from chicken feathers, and the highest yield was 94%, while the yields of keratin through sodium sulfide and L-cysteine were about 88% and 66% in another study [105]. Ramya et al. [76] extracted keratin from red sheep’s hair using sodium metabisulfite, urea and sodium dodecyl sulfate (SMBS). This method had a yield of 96%, but the high cost for operation and chemicals, highly concentrated agents, long processing time, and potential health and environmental risks limited its application in industrial production. Therefore, Tasaki [77] developed a two-step thermal hydrolysis process without the use of chemicals to extract keratin from hog hair, and the yield was nearly 70%.

### 3.6. Chitin/chitosan

Chitin and chitosan are commonly found in crustaceans, insects, and microorganisms [43]. Among them, the shells of crustaceans such as shrimp, crab, and lobster are the most significant sources. Through enzymatic or chemical deacetylation, chitin can be converted to its derivative, chitosan. Due to their hydroxyl, amino and carbonyl groups, they can be acylated, esterified, etherified, alkylated, oxidized, chelated, graft copolymerized, and crosslinked. Both chitin and chitosan are widely used in biomedicine, water treatment, enzyme immobilization, stabilization, purification, and so on [15,106,107].

The most common methods to isolate chitin are chemical and biological treatments that involve two steps, demineralization and deproteinization. Concentrated acids and bases are usually used in chemical treatments, while microorganisms including lactic acid bacteria and other microbial species are applied in biological route [108]. In recent years, these methods have been modified to reduce operation time, and improve the purity and thermal stability of chitin and chitosan. Kaya et al. [78] reported that repeating a sodium hypochlorite (NaClO) treatment twice for 10 min before demineralization and deproteinization could speed up the isolation of chitin from the shells of crab, crayfish and shrimp, and save energy. In addition, the percentage chitin yields from biomass wastes through this new method were in the range of 13–14%, which were very similar to the previously reported yields by conventional way (10–20%). Eddya et al. [43] investigated the effect of acid concentration on the chitosan extraction efficiency from shrimp shell. The result showed that chitosan obtained from concentrated acid and base treatment was highly pure with the degree of deacetylation of 80% and had better heat resistance compared with commercial chitosan. Besides, chitin nanocrystals were also obtained from prawn shell (20% yield) [109], oceanic biomass wastes [110] and shrimp [111] by extraction and acid hydrolysis. These nanocrystals were then applied as Pickering emulsion stabilizers, and functional additives in bio-nanocomposites to enable good water treatment capacity, and antibacterial and antioxidant activities.

## 4. Functional materials derived from biomass wastes

In spite of being good sources of natural polymers, biomass wastes also have functional groups and unique properties, so they can be directly reused, especially as adsorbents and construction materials [112,113]. In addition, they can be used as precursors or base to make functional carbon-based materials through pyrolysis and hydrothermal, physical, and chemical activations, which has attracted much attention in last five years.

Till now, various forms of carbon-based materials, including biochar, activated carbon (AC), graphitic carbon, and so on, have been developed. Both biochar and AC are produced by pyrolysis, but there are fine distinctions between them, such as feedstock, pyrolysis conditions, activation treatments, porous structures, and applications [114]. The feedstock for biochar includes various biomass wastes, such as peanut shell, corn stalk, and some animal wastes. Biochar is obtained by the pyrolysis of organic materials at the temperature of below 700°C and limited oxygen conditions in a hot sealed reactor [115]. This process is eco-friendly, available, and efficient compared to the productions of other charcoals because the reactors are nontoxic and recyclable. The pyrolysis conditions, such as the temperature and ramping rate, have significant impact on the microstructures of biochar, resulting in various functional properties [116]. Therefore, biochar has shown great potentials in water treatment, soil amendment, and catalytic oxidation of organic pollutants [115]. However, if pyrolysis process is incomplete, pollutants such as NO_x_, SO_x_, smoke, aerosols, and unburned hydrocarbons will be formed and released, impacting environment and health [117]. AC can be produced from biomass or other carbonaceous substances (e.g. coal) through pyrolysis at a temperature above 700°C. AC usually has larger specific surface area and smaller microporous structure compared to biochar. It is worth noting that biochar can be converted into AC with optimized porous structure and specific surface area by activation process (e.g. KOH and NaOH chemical and physical activation). Thus, the biochar-based AC was prepared and applied in electrochemical double-layer capacitors (EDLC) [16].

Graphitic carbon is commonly obtained from soft carbons (e.g. petroleum coke) via heating above 2100°C [118]. The in-plane structure of graphene layers in the graphitic carbon is almost similar to that in graphite. It is produced by various synthetic methods to increase the degree of graphitization, including direct heating of porous carbons at 2500–3000°C, and catalytic graphitization where in-situ graphitic nanostructure was obtained by metal catalysts [119].

**Figure 2.**
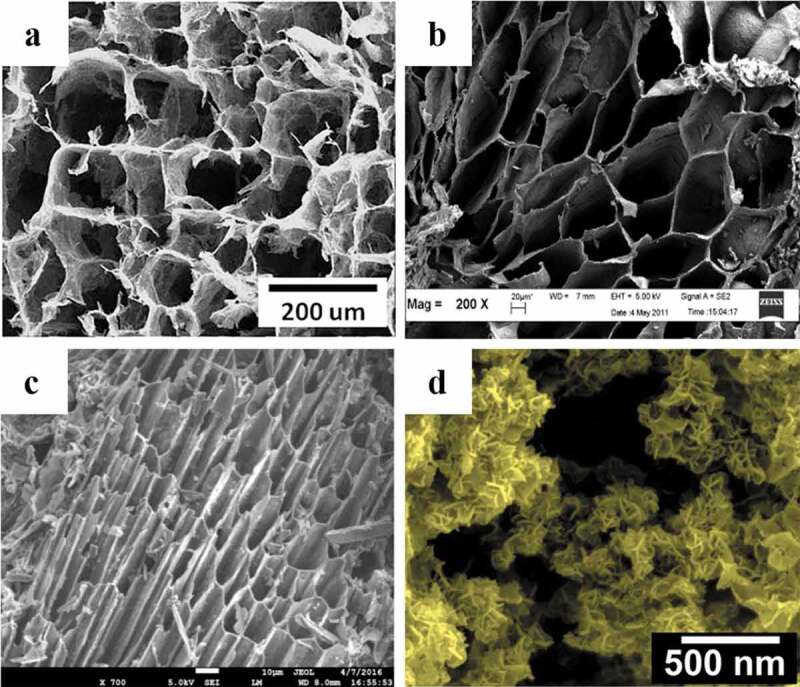
SEM images of carbon-based materials derived from biomass wastes: (a) lignin-modified graphene aerogel from corncob; (b) activated carbon from rambutan peel; (c) biochar from wheat straw; and (d) graphitic-carbon nanoflakes from green tea waste [2,97,121,122] (Reproduced by permissions from [2], copyright [2016, Elsevier], from [97], copyright [2018, Elsevier], from [121], copyright [2014, Elsevier] and from [122], copyright [2019, Elsevier])

These carbon-based materials not only possess high specific surface area, porous structure, and abundant surface functional groups, but also exhibit favorable chemical stability, great performance, and regeneration capacity [120]. Their scanning electron microscopy (SEM) images are shown in Figure 2. Many efforts have been made to investigate their potential applications as adsorbents, catalyst carriers, electrode materials, and functional composites.

### 4.1. Adsorbent materials

Functional groups including carboxyl, hydroxyl, sulfhydryl, and amide of biomass wastes play an important role in adsorbing contaminants from liquid phase or natural gas. The adsorption of heavy metals by directly utilizing the raw biomass wastes, such as leaves [112], orange peel [8], tea waste [123], crop shells [113], and eggshell membrane (ESM) [124], has been studied. It can not only lower the production cost of adsorbents but also improve the recovery efficiency of biomass wastes. Lee and Choi [125] used raw persimmon leaves (RPL) and dried persimmon leaves (DPL) as adsorbents to remove heavy metals (Pb^2+^, Cu^2+^ and Cd^2+^), because persimmon leaves have numerous hydroxyl groups that can bind with heavy metals. The study revealed that both RPL and DPL displayed high removal efficiencies (over 98%) towards Pb^2+^, Cu^2+^ and Cd^2+^ at the concentrations of adsorbates below 1 mg/L. Compared to RPL, the removal rate of DPL was 10–15% higher, and the removal efficiency of Pb^2+^ was highest among three heavy metals, followed by Cu^2+^ and Cd^2+^. In terms of adsorption performance, the maximum adsorption capacity of Pb^2+^, Cu^2+^ and Cd^2+^ was 22.59 mg/g, 19.42 mg/g, and 18.26 mg/g, respectively. Monteiro et al. [126] reported that seafood waste, crab carapace and clam shell, could be used to remove Hg^2+^ and Cd^2+^ from wastewater, and the Hg^2+^ removal efficiency of both adsorbents were higher than 80% in monometallic solutions, while the uptake of Hg^2+^ was limited due to high kinetic and equilibrium selectivity for Cd^2+^ in binary solutions. Feizi and Jalali [113] investigated competitive adsorption of heavy metals (Cd^2+^, Cu^2+^, Ni^2+^, Zn^2+^, Fe^3+^, and Mn^2+^) by sunflower, potato, canola, and walnut shell. The study showed that the adsorption capacities of biosorbents were as followed: sunflower > potato > canola > walnut shell. Among various heavy metals, the adsorption capacity towards Cd^2+^ was highest, up to 50–70 mg/g at the concentration of adsorbate of around 150 mg/L. ESM also has good adsorbent properties for the removal of heavy metals and dyes from wastewater. Choi [124] esterified carboxylic groups to endow ESM with a high cationic charge density, and thus improved the capacity to remove negatively charged sulfur dye. The results revealed that methyl-esterified ESM showed over 98% removal of sulfur dye at optimal condition, and 0.68–0.73 mg/L sulfur dye could be adsorbed by 1 mg/L absorbent.

However, there are some drawbacks of directly using raw biomass wastes as adsorbents. For example, the removal rate of heavy metals is relatively low due to the slow diffusion or limited surface-active sites. It is also difficult to separate the absorbents from solutions and recycle them for multiple usage. As a result, the conversion of biomass wastes into different forms of carbon-based materials with better adsorption capacities has been investigated in the past five years.

Biochar is widely used as adsorbents for removing heavy metals from wastewater. Yu et al. [2] made three kinds of biochars from peanut shell, corn stalk and wheat straw to remove hydrophilic ionic liquid, 1-butyl-3-methyl-imidazolium chloride ([BMIM][Cl]). It was found that these biochars exhibited similar microporous structures and a large number of oxygen-containing functional groups. All of them showed higher removal rates (q_max_ of 0.644 ~ 0.888) than those of other reported carbon-based materials (e.g. q_max_ of 0.170 ~ 0.520). As well, Sattar et al. [127] used peanut shell as feedstock material for the production of biochar to remove As^5+^ and As^3+^ from water. The As^5+^ and As^3+^ removal rates of peanut shell biochar (PSB) were up to 99% (at pH 6.2) and 95% (at pH 7.2) with 0.6 g/L adsorbent dose, 5 mg/L initial As ion concentration, and 2 h equilibrium time, which was due to its abundant surface functional groups. In order to further improve the adsorption capacity, biochars are modified with special functional groups through chemical treatments. Chen et al. [128] prepared phosphoric acid-modified biochar (CFCP) from chicken feather, whose functional groups and super surface area were beneficial to its adsorption capacities of Cd^2+^ and Pb^2+^. The results showed that, compared to the biochar without modification, CFCP had faster adsorption rate probably due to the increased N-containing heterocycles. Gao et al. [129] prepared biochar from grapefruit endothelium and modified it by iron ion and polyaniline through biosorption-pyrolysis method. This novel biochar displayed superior removal capacity towards Cr^6+^ (up to 100% within 135 min at initial Cr^6+^concentration of 50 μM, 30 mL) mainly due to the synergistic effect of polyaniline and iron ion. Peng et al. [130] obtained biochars from corn stalk, almond shell, and dairy manure with Fe/Al (hydr)oxides via co-precipitation. These biochars displayed better performance for removing phosphate than other adsorbents possibly due to the synergistic effects of Fe/Al oxides and biochar with larger surface area, higher pore volume, and more reactive surface hydroxyl sites. However, the application of biochar in water treatment still faces the struggle of the inconvenient separation [115].

Biomass-derived AC also can be used as adsorbents for environmental remediation. The adsorption capacity of AC usually depends on the precursor, activation methods, physical and chemical pre-treatments, type of activating agents, and gasification time [131]. Especially, after thermal or chemical pre-treatments, the surface functional groups and physical and chemical structures of AC can be improved, resulting in the better adsorption performance. Mehrvarz et al. [131] generated AC from the broom sorghum stalk, followed by the treatment with triethylenetetramine (TETA) to functionalize the surface of AC. The study showed that the modified AC dramatically enhanced the selectivity of CO_2_/CH_4_ and the adsorption of CO_2_ was up to 3.20 mmol/g. Njoku et al. [121] used microwave heating for KOH activation to obtain AC from rambutan peel, which was different from conventional heating methods. This method could shorten the activation time significantly (12 min for this study but 60 min for others) and the adsorption capacity of the resultant AC for acid yellow 17 dye was in the range of 49.14 and 215.05 mg/g at dye concentrations from 50 to 400 mg/L. Georgin et al. [35] investigated the adsorption capacity of AC derived from peanut shell through conventional pyrolysis and microwave irradiation-pyrolysis (MW-P). It was found that AC obtained by MW-P presented stronger adsorption capacity for organic dyes at pH 2.5 due to its higher total pore volume (0.210 cm^3^/g) and larger surface area (395.80 m^2^/g) compared to the conventionally pyrolyzed samples. The adsorptions towards direct black 38 and reactive red 141 dyes were between 56.7 and 96.6 mg/g and 165.7–260.3 mg/g, respectively, at the concentrations of dyes of 100–350 mg/L.

Overall, the direct use of biomass wastes as adsorbents is a simple and cost-effective way to remove the heavy metals. Through modifying the structure or surface functional groups of biomass wastes, the adsorption capacity could be improved. Additionally, carbon-based adsorbents obtained from biomass wastes usually have higher adsorption capacities due to their larger surface area and higher porosity. However, these processes increased the cost, and the recyclability of absorbents needs to be improved.

### 4.2. Catalytic materials

The conversion of biomass wastes into value-added biochar as catalysis matrix is another attractive option. Liu et al. [115] studied the performance of nitrogen-doped magnetic biochar produced from rice straw in catalytic oxidation of metolachlor. The study showed that the doped nitrogen atoms could significantly increase the surface basicity and facilitate the catalytic degradation of metolachlor along with peroxymonosulfate. In addition, adding proper transition metals into biomass-derived biochar has been illustrated to be beneficial to endow it with good catalytic capacity. Liu et al. [132] explored a one-pot method to make an Ag loaded biochar hybrid material via fast pyrolysis from the fir sawdust and studied its catalytic capacity of the HCOOH-induced Cr^6+^ reduction. Ag@biochar could facilitate the reduction from Cr^6+^ to Cr^3+^ in liquid within 20 min with the efficiency of 93%, which revealed its good catalytic activity to this reaction. Considering the adverse environmental effect on land caused by uncontrolled release of caffeine in spent coffee ground, Cho et al. [133] fabricated cobalt (Co)-loaded waste coffee grounds to produce value-added Co-biochar catalyst in one-step. It was found that the catalyst presented superior catalytic capacity to reduce 70.1% of 0.17 mM PNP within 5 min.

**Figure 3.**
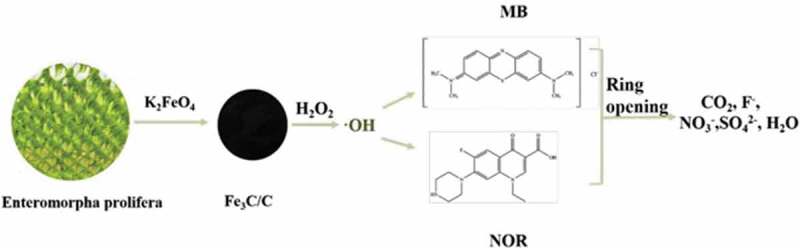
Schematic diagram of Fe_3_C/C composite for methylene blue removal and NOR degradation [134] (Reproduced by permission from [134], copyright [2019, Elsevier])

AC is another option as a catalyst support due to its great internal surface area, high inertness, and versatility. However, biomass waste-derived AC usually displays the low recoverability and reutilization. Thus, Huang et al. [134] chose *Enteromorpha prolifera* (a kind of marine green alga) and K_2_FeO_4_ as bases to fabricate Fe_3_C/C composite (an improving Fenton-like catalyst) via a simple one-step calcination synthesis method (Figure 3). The results revealed that the catalyst exhibited great catalytic and adsorption capacities, which could decrease the chemical oxygen demand (COD) volume (below 50) in actual plant wastewater and be catalytic for antibiotic norfloxacin (NOR) degradation. It also presented perfect stability and recyclability.

### 4.3. Electrode materials

Over the past few years, many researches have focused on converting biomass wastes into energy-storage materials. The carbon-based materials derived from biomass wastes with high specific surface area can facilitate the process of interfacial interactions between the electrode and electrolyte ions by decreasing ions delivering resistance and diffusion distance [119]. Table 2 shows the specific properties of electrode materials derived from different biomass wastes.

**Table 2.** Electrode porous carbons derived from biomass wastes

Raw Material	Treatment	Electrolyte	Specific capacitance
Rice husks [135]	KOH activation, at temperatures between 400 and 900°C	6 M KOH	367 F/g at 5 mV/s*
		1.5 M tetraethylammonium tetrafluoroborate (TEA-BF_4_) in acetonitrile	174 F/g at 5 mV/s*
Loblolly pine chips [16]	Different carbonization methods and NaOH activation	6 M KOH	74 F/g at 20 mV/s*
Tissue paper produced by wood pulp [136]	One-step carbonization and activation treatment	6 M KOH	~200 F/g at 1 mV/s*
Tissue paper produced by straw [136]			~150 F/g at 1 mV/s*
Dawn redwood cone [137]	Pre-carbonization and chemical activation	6 M KOH	197 F/g at 1.0 A/g*
Starch-based packing peanuts [119]	KOH activation	1 M TEA-BF_4_ in acetonitrile	149 F/g at 0.5 mA/cm^2^*
Cherry stones [138]	Pretreatment of lignin dissolution-precipitation	6 M KOH	370.5 F/g at 0.5 A/g**
Palm-shell [139]	Graphitic activated carbon prepared by dispersion of AC in the graphene layers	1 M HCl	54.6 F/g**
Green tea waste [140]	KOH activation with water or hydrochloric acid treatment	1 M H_2_SO_4_	~162 F/g at 0.5 A/g**

*Tested in two-electrode system **Tested in three-electrode system

EDLC and pseudocapacitors are two device schemes of supercapacitors. However, the applications of pseudocapacitor are limited because of their poor cycle stability and low electrical conductivity [138]. Due to the high-power density and superior charge-discharge stability of EDLC, more studies are focusing on them. Generally, different forms of carbon-based electrode materials include AC [135], biochar-based AC [16], graphitic carbon [119], microporous carbons [136], and so on. Gao et al. [135] transformed rice husk into AC through KOH activation. Because SiO_2_ nanocrystals were encompassed by a carbon matrix in the rice husk, the AC kept the size and shape, and had an increased ordering degree of carbon which showed great high-power solving performance and electrochemical cycle capacity. Moreover, He et al. [16] recycled loblolly pine chips to fabricate biochar-based ACs by NaOH activation and then assembled it into EDLCs as electrode materials due to its high surface area and huge pore volume.

Many factors have the impact on the electrochemical capacity such as the structure of raw materials, carbonization temperature, activation and pretreatments, which lead to various pore structures, high specific surface area (SSA) and high porosity of carbon materials [136]. For example, two kinds of tissue papers (from wood pulp and straw) were converted into carbon materials by one-step carbonization and activation treatment. It was revealed that the carbon with microporous structure obtained from wood pulp at 700°C displayed better capacitance (~200 F/g at 1 mV/s), while the carbon derived from straw presented hierarchical porous structure with decreased capacitance at 900°C [136]. Jia et al. [137] prepared nitrogen-doped porous carbon from dawn redwood waste through carbonization combination with chemical activation. It displayed a large SSA of 1831 m^2^/g and superior performance with the specific capacitance of 326 F/g at current density of 0.5 A/g. Zhang et al. [138] fabricated the nitrogen-doped carbon from cherry stones which showed hierarchical porous structure through the lignin dissolution-reprecipitation pretreatment and molten salt activation. It was found that the material possessed the excellent specific capacitance of 370.5 F/g at the current density of 0.5 A/g, and about 99% of this capacitance was kept after 5,000 cycles.

Among these carbon-based electrode materials, graphitic carbon has been considered as a promising electrode material due to its low cost, superior electrochemical stability, large SSA (1000–3000 m^2^/g), high specific capacitance and eco-friendliness [122]. Sankar et al. [122] successfully prepared ultrathin mesoporous graphitic carbon from green tea via KOH activation, with either water or hydrochloric acid treatments. It was shown that water-treated graphitic-carbon nanoflakes presented higher specific capacitance of 162 F/g at 0.5 A/g.

It is worth noting that electrode materials can also be used to treat wastewater through capacitive deionization (CDI). Chong et al. [139] prepared graphitic-activated carbon (GAC) from palm-shell, which displayed high electrical conductivity and better electrosorption stability compared to AC electrodes.

### 4.4. Composite materials

In general, two or more components that possess distinctive properties can consist of composite materials, and the properties of composites are not attainable with the separated components [141]. In recent years, the use of biomass wastes like rice husk [25], rice straw [142], flax [143], and fly ash [23], as unconventional construction materials are an interesting solution, since the construction department should always face the issues about greenhouse gas emission, the disposal and recycling of raw materials and sustainable development [25]. Hu et al. [23] prepared reactive rice husk ash using the self-designed combustion system and applied it in cement-base materials. It was found that the compressive strength of motors was improved with the increase of the rice husk ash content. As well, this approach to utilize rice husk ash was environmentally friendly, which was indicated by sustainability analysis. Martinez-Lage et al. [10] also reported that with the addition of biomass ash, the flexural strength decreased, but the compressive strength of all the composite mortars increased.

**Figure 4.**
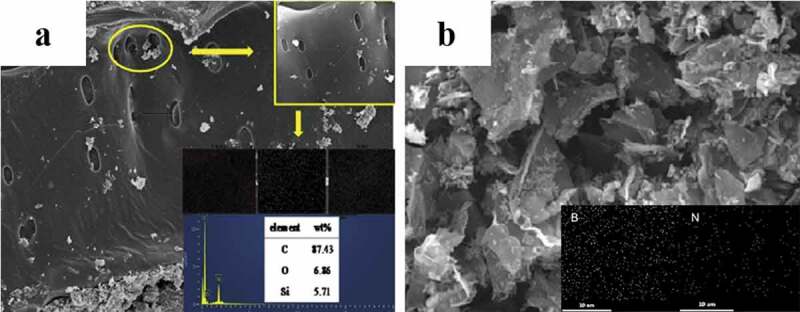
SEM images and elemental mapping of composite materials derived from biomass wastes: (a) silica-loaded biochars from bamboo; and (b) nitrogen and boron dual-doped aerogel from pomelo peel [146,148] (Reproduced by permissions from [146] and [148], copyright [2019, Elsevier])

Despite of the construction applications, the carbon-based materials derived from biomass wastes were also widely used to endow the composites with other functional properties, such as conductivity, adsorption, hydrophobicity, and so on. Zhu et al. [144] developed an interfacial catalytic engineering protocol to prepare carbon microtube anodes for Li batteries that were graphitic, porous and co-doped with heteroatoms from hair waste. During the biomass graphitization, Ni-based nanofilm played an important catalytic role in Li^+^ diffusions and formation of deep pores. It was revealed that the microtube presented superior anodic behaviors on recycling capacity, active utilization efficiency, long-term cyclic stability, and rate capabilities. However, the complicated preparation and the existence of toxic and non-sustainable chemicals limit its application [145]. Hence, the interlayer is proposed, which is a carbon film placed between the separator and sulfur cathode to improve the rate ability and cyclic stability in Li-S batteries [145]. The introduction of interlayer is beneficial to reuse the active compounds during energy storage. Zhu et al. [146] prepared nitrogen and boron dual-doped aerogel (NB-PPCA) from pomelo peel by hydrothermal process, freeze-drying, and pyrolysis process (Figure 4). It was used as an interlayer on pristine separator in Li batteries, and showed superior initial discharge capacity, high specific capacity, and better cyclic stability and rate ability compared to other cells with PPCA separator and pristine separator.

In terms of adsorption capacity enhancement, apart from chemical modification, the incorporation of nanoparticles is another effective way. Li et al. [147] assembled nano-hydroxyapatite (nHAP) and wood-processing residues, wheat straw and Chinese medicine residues to prepare nHAP@biochar. The nHAP particles had a large surface area, and the calcium on their surface could be easily replaced by heavy metals. It was found that tylosin and Cu^2+^ could be removed by nHAP@biochar simultaneously but the adsorption quantities for the antibiotic and heavy metal were affected by the pyrolysis temperature. Zhao et al. [148] reported the effect of adding silica particles in bamboo-derived biochar on its stability and adsorption capacities for tetracycline (Figure 4). The total pore volume, thermal stability, and adsorption capacity of biochar obviously increased after adding silica, which was attributed to the structure of silica and interaction between silica and biochar. Fan et al. [149] fabricated a magnetic tea waste/Fe_3_O_4_ composite via a chemical co-precipitation method. Loading tea waste with nano-Fe_3_O_4_ particles not only could enhance the stability of nanoparticles but also endowed the material with excellent superparamagnetic property for easy separation. The chromium adsorption test revealed that this composite presented high adsorption capacity of up to 75.76 mg/g. It also had a good reusability, and the removal rate was still higher than 70% after five recycling cycles.

The hydrophobic property of composite is worth noting as well because it is relevant to the functionalities such as catalytic property and self-cleaning. Chen et al. [97] modified graphene aerogel by mixing lignin from corncob through one-step hydrothermal treatment. This kind of aerogel showed good capacity for adsorbing petroleum oils and toxic solvents (e.g. toluene, chloroform, carbon tetrachloride), owing to the hydrophobic constituents in lignin skeletons and porous structure of graphene aerogels. Besides, the aerogel could be reused by repeated heat treatment and compression. Fitria et al. [150] used coffee bean waste to produce hydrophobic layer, which was obtained through carbonization method, dispersed in acetic acid, and then coated with polyvinyl alcohol (PVA) binder. It was shown that the ratio of carbon and PVA could affect the hydrophobicity property. The composite possessed comparable hydrophobicity compared to that of other well-known materials.

## 5. Conclusion and future perspectives

This review highlights the research work on the biomass waste reuse and recycling in recent five years, where agricultural and forestry residues, animal wastes, industrial wastes, and municipal solid wastes have been frequently studied. Instead of treating these biomass wastes in traditional methods (e.g. incineration and landfill), many researchers intended to extract natural polymers such as cellulose, lignin, gelatin, chitin and so on from biomass wastes through various approaches for further applications in food, agriculture, and medicine. Compared to conventional extraction approaches, novel methods such as organosolv process (especially two-phase system), combination of mechanical, chemical and biological treatments, and incorporation of pressure or ultrasonication have been proposed to isolate natural polymers in a more efficient and eco-friendly way. These technologies also aim to improve the yields and purities of extracted products, simplify the extraction processes, and enhance the functional properties of extracts. Several efforts have been made to directly reuse biomass wastes as adsorbents for water contaminant removal or additives of composite materials in a simple and cost-effective way, but more attention has been paid to convert them into carbon-based materials (e.g. biochar, AC, and graphitic carbon) with large surface area, porous structure, favorable chemical stability, great performance, and regeneration capacity. Various methods such as thermal and chemical pretreatments and incorporation of nanoparticles have been applied to further improve the functionalities of carbon-based materials so as to enable their applications as adsorbents, catalyst carriers, electrode materials, and functional composites.

It is believed that biomass wastes are a promising source of natural building blocks, and the reuse and recycling of biomass wastes could relieve the environmental pressure. However, future research in the following areas is required to promote their practical applications:

Quality control is the key to future development. Current biomass wastes derived products are prepared in research labs, so it is easy to get enough raw materials from the certain suppliers. Once the production scale is enlarged, the required biomass wastes collected from different seasons and locations may vary. Therefore, the quality control is necessary to determine the compositions of biomass wastes and the molecular structure of targeted natural polymers, so as to ensure the desirable properties of final products.

Feasible and cost-effective approaches for the conversion of biomass wastes into value-added products are expected in future studies. For the extraction of natural polymers, more works should focus on the development and optimization of extraction methods that are able to scale up and can generate products with high yields and purities for each major biomass wastes. In terms of functional materials derived from biomass wastes, systematically study is needed to better modulate the properties of carbon-based materials by actively designing and controlling their structures. Moreover, a wider vision should be encouraged to develop other types of materials (rather than carbon) so as to facilitate the reuse and recycling of biomass wastes.

Safety issues of biomass wastes derived products need to be investigated. Although the major components in biomass wastes are biocompatible and nontoxic, there are still certain amounts of ‘impurities’ or contaminants existed. Moreover, various nanoparticles are incorporated to improve the performance of composite materials, which also cause some concerns. Therefore, it is necessary to study case-by-case the fate of these minorities in materials and their potential safety issues.
